# *Stenoxylitaquadrifasciata* sp. nov. (Coleoptera, Melandryidae) from Guizhou, southwest China

**DOI:** 10.3897/BDJ.12.e125966

**Published:** 2024-07-15

**Authors:** Anzhi Dang, Shulin Yang

**Affiliations:** 1 Guizhou Institute of Environmental Sciences Research and Design, Guiyang, China Guizhou Institute of Environmental Sciences Research and Design Guiyang China; 2 School of Life Sciences, Guizhou Normal University, Guiyang, China School of Life Sciences, Guizhou Normal University Guiyang China

**Keywords:** false darkling beetles, melandryid, Leigongshan, Queniao Village, Getou Village

## Abstract

**Background:**

The genus *Stenoxylita* Nomura, 1959 (Coleoptera, Melandryidae) currently contains two species, *Stenoxylitatrialbofasciata* (Hayashi & Katö, 1956) from Japan and Shaanxi, China (Konvička 2015) and *Stenoxylitasasajii* Toyoshima, 2001 from North Vietnam. We discovered a new species of the genus from the Mountain Leigong of Guizhou Province, China.

**New information:**

A new species, *Stenoxylitaquadrifasciata* **sp. nov.**, is described from Mountain Leigong, Leishan County, Guizhou Province of southwest China. It is the third species of this genus and the second species of this genus from mainland China.

## Introduction

The genus *Stenoxylita* was established by Nomura (1959) with the designation of *Dircaeomorphatrialbofasciata* Hayashi & Katö, 1956 as the type species. The genus is similar to the genus *Xylita* Paykull, 1798, but differs from the latter in the unemarginated eyes, the pronotum with a median longitudinal impression, unbordered sides of apical pronotum and dilated protarsomeres I - III (Nomura 1959). This genus belongs to the tribe Dircaeini Kirby, 1837 of the subfamily Melandryinae Leach, 1815 (Nikitsky and Pollock 2010). Toyoshima (2001) described the 2^nd^ species of this genus, *Stenoxylitasasajii*. Currently, there are only two known species of the genus, *Stenoxylitatrialbofasciata* (Hayashi and Katö 1956) from Japan and Shaanxi, China (Konvička 2015) and *Stenoxylitasasajii* Toyoshima, 2001 from North Vietnam.

We collected several specimens from Queniao Village and Getou Village of the Mountain Leigong area, Leishan County, Guizhou Province, China and identified them to this genus. These specimens are characterised by the four disconnected pale bands on each elytron, which differ from the two known species. We describe them here as *Stenoxylitaquadrifasciata* sp. nov. based on the four elytral bands. It is the second species of this species discovered in China.

## Materials and methods

Specimens were collected using flight intercept traps baited with ethanol. Collected specimens were either pinned directly or glued on to pinned paper cards. All materials are preserved at the School of Life Sciences, Guizhou Normal University, Guiyang, China (GZNULS).

An Olympus SZ61TR stereomicroscope was utilised for specimen observation and dissection. Photos of the adult habitus were captured using a Canon EOS R6 II digital camera paired with a Canon RF 100 mm lens.

## Taxon treatments

### Stenoxylita quadrifasciata

Dang and Yang, 2024 sp. nov.

48288AA9-2206-59DC-8E01-18EEA3717171

E1D7F9C7-0FA3-4C6B-8292-E177D9AFCC01

#### Materials

- **Type status:** Paratype. **Occurrence:** recordedBy: Shulin Yang; individualCount: 1; sex: female; lifeStage: adult; occurrenceID: FB6BC911-9ED4-5C89-ACF9-30A5A4FF9F52; **Taxon:** scientificName: Stenoxylitaquadrifasciata; **Location:** country: China; stateProvince: Guizhou; county: Leishan; locality: Queniao Village; verbatimCoordinateSystem: decimal degrees; verbatimSRS: WGS84; decimalLatitude: 26.401; decimalLongitude: 108.2261; geodeticDatum: https://epsg.io/4326; **Event:** samplingProtocol: flight interception trap; year: 2019; month: 5; day: 5
- **Type status:** Paratype. **Occurrence:** recordedBy: Shulin Yang; individualCount: 1; sex: female; lifeStage: adult; occurrenceID: 4467B291-55E7-5936-9F2E-A6DDC3A8A21B; **Taxon:** scientificName: Stenoxylitaquadrifasciata; **Location:** country: China; stateProvince: Guizhou; county: Leishan; locality: Getou Village; verbatimCoordinateSystem: decimal degrees; verbatimSRS: WGS84; decimalLatitude: 26.3917; decimalLongitude: 108.2366; geodeticDatum: https://epsg.io/4326; **Event:** samplingProtocol: flight interception trap; year: 2019; month: 5; day: 5
- **Type status:** Paratype. **Occurrence:** recordedBy: Shulin Yang; individualCount: 1; sex: female; lifeStage: adult; occurrenceID: 1858B59B-ECC1-5315-B5C6-5BCCD18629EF; **Taxon:** scientificName: Stenoxylitaquadrifasciata; **Location:** country: China; stateProvince: Guizhou; county: Leishan; locality: Queniao Village; verbatimCoordinateSystem: decimal degrees; verbatimSRS: WGS84; decimalLatitude: 26.4014; decimalLongitude: 108.2249; geodeticDatum: https://epsg.io/4326; **Event:** samplingProtocol: flight interception trap; year: 2017; month: 5; day: 5
- **Type status:** Paratype. **Occurrence:** recordedBy: Shulin Yang; individualCount: 1; sex: female; lifeStage: adult; occurrenceID: 92D43783-0AF1-5888-9E50-99253C3D4775; **Taxon:** scientificName: Stenoxylitaquadrifasciata; **Location:** country: China; stateProvince: Guizhou; county: Leishan; locality: Getou Village; verbatimCoordinateSystem: decimal degrees; verbatimSRS: WGS84; decimalLatitude: 26.3908; decimalLongitude: 108.2347; geodeticDatum: https://epsg.io/4326; **Event:** samplingProtocol: flight interception trap; year: 2019; month: 5; day: 5
- **Type status:** Paratype. **Occurrence:** recordedBy: Shulin Yang; individualCount: 1; sex: female; lifeStage: adult; occurrenceID: DD18643A-D448-59B4-80B5-AFFBD0D12EB5; **Taxon:** scientificName: Stenoxylitaquadrifasciata; **Location:** country: China; stateProvince: Guizhou; county: Leishan; locality: Getou Village; verbatimCoordinateSystem: decimal degrees; verbatimSRS: WGS84; decimalLatitude: 26.3917; decimalLongitude: 108.2366; geodeticDatum: https://epsg.io/4326; **Event:** samplingProtocol: flight interception trap; year: 2019; month: 5; day: 5
- **Type status:** Paratype. **Occurrence:** recordedBy: Shulin Yang; individualCount: 1; sex: female; lifeStage: adult; occurrenceID: 7AC7B779-5D5C-5B12-BE54-24CD5E92E5B7; **Taxon:** scientificName: Stenoxylitaquadrifasciata; **Location:** country: China; stateProvince: Guizhou; county: Leishan; locality: Queniao Village; verbatimCoordinateSystem: decimal degrees; verbatimSRS: WGS84; decimalLatitude: 26.391; decimalLongitude: 108.2353; geodeticDatum: https://epsg.io/4326; **Event:** samplingProtocol: flight interception trap; year: 2015; month: 5; day: 7
- **Type status:** Paratype. **Occurrence:** recordedBy: Shulin Yang; individualCount: 1; sex: female; lifeStage: adult; occurrenceID: 56C77E20-E152-54CA-BB37-9AB4173F3FEB; **Taxon:** scientificName: Stenoxylitaquadrifasciata; **Location:** country: China; stateProvince: Guizhou; county: Leishan; locality: Queniao Village; verbatimCoordinateSystem: decimal degrees; verbatimSRS: WGS84; decimalLatitude: 26.4018; decimalLongitude: 108.2246; geodeticDatum: https://epsg.io/4326; **Event:** samplingProtocol: flight interception trap; year: 2019; month: 5; day: 5
- **Type status:** Paratype. **Occurrence:** recordedBy: Shulin Yang; individualCount: 1; sex: female; lifeStage: adult; occurrenceID: D4C7DB93-C4F8-5D4C-AC07-02577E1F1E0B; **Taxon:** scientificName: Stenoxylitaquadrifasciata; **Location:** country: China; stateProvince: Guizhou; county: Leishan; locality: Queniao Village; verbatimCoordinateSystem: decimal degrees; verbatimSRS: WGS84; decimalLatitude: 26.391; decimalLongitude: 108.2353; geodeticDatum: https://epsg.io/4326; **Event:** samplingProtocol: flight interception trap; year: 2017; month: 5; day: 5
- **Type status:** Paratype. **Occurrence:** recordedBy: Shulin Yang; individualCount: 1; sex: female; lifeStage: adult; occurrenceID: FA93297E-35F1-5338-8A55-97CE39AD49BE; **Taxon:** scientificName: Stenoxylitaquadrifasciata; **Location:** country: China; stateProvince: Guizhou; county: Leishan; locality: Queniao Village; verbatimCoordinateSystem: decimal degrees; verbatimSRS: WGS84; decimalLatitude: 26.391; decimalLongitude: 108.2353; geodeticDatum: https://epsg.io/4326; **Event:** samplingProtocol: flight interception trap; year: 2017; month: 5; day: 5

#### Description

**Figure 1.**
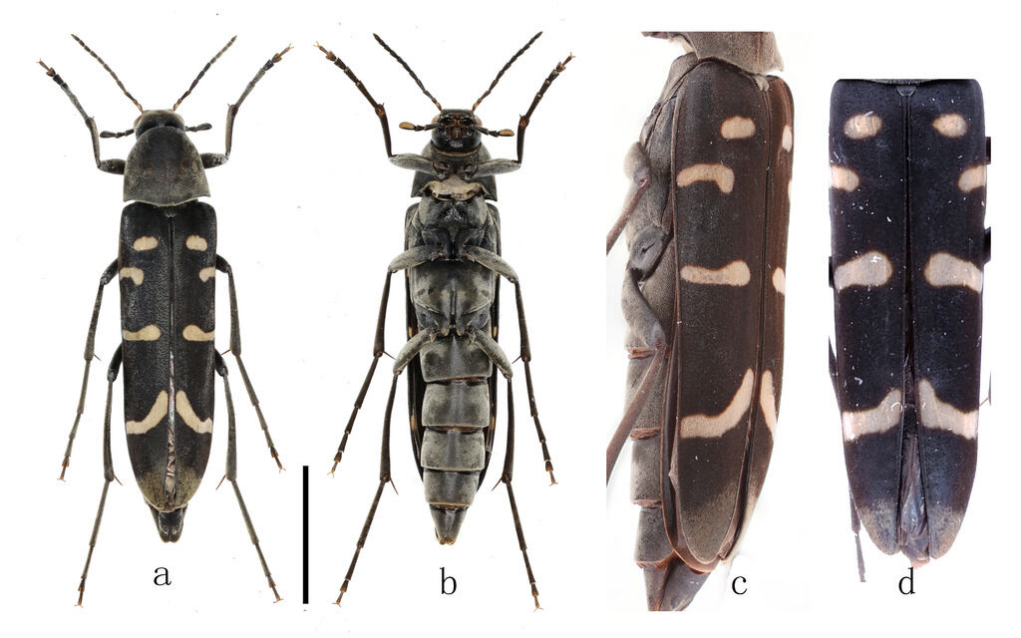
Habitus of *Stenoxylitaquadrifasciata* sp. nov. females, a. dorsal view; b. ventral view; c. dorsolateral view of body; d. variation of elytral bands. (a, b, scale bar, 5 mm, c, d, not to scale).

**Figure 2.**
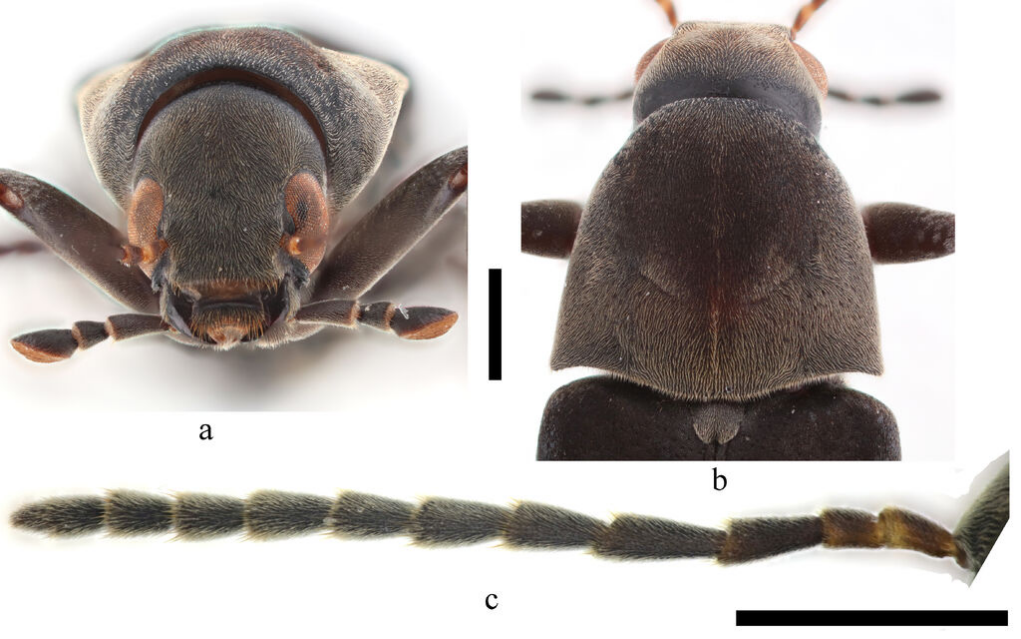
Habitus of *Stenoxylitaquadrifasciata* sp. nov. female, a. head, front view; b. pronotum, dorsal view; c. antennae, left, dorsal view; (scale bars, 1 mm).

**Adults**. Female (Figs 1, 2). **Body** oblong ovate, length 10.2 – 14.3 mm, width at maximum level of elytra 2.5 – 3.5 mm (n = 9), black, except epistoma, apical surface of maxillary palpi and four bands on each elytron yellowish-white, first two antennomeres dark brown and dorsal apices of them yellowish-white. Body covered with fine pubescence, colour paler on part of head, pronotum, femur, tibia, elytral bands and apical 1/6 of elytra. **Head** (Fig. 2a) small, finely punctured. Maxillary palpi long, subcylindrical, second segment longest, slightly thickened apically, third short, obliquely obconical, fourth obliquely truncated. Frons raised, nearly square, sides slightly concave at antennal insertions. Eyes large, elongate oval, weakly emarginate behind antennal insertions. Antennae (Fig. 2c) short and slender, generally reach base of pronotum, first antennomere longer than second, third longer than second, fourth the longest, gradually shortened from fifth to tenth, each antennomere of third to tenth weakly dilated apically, eleventh pointed apically, longer than tenth, ratios of antennomeres about 1:0.7:1.3:1.6:1.4:1.2:1.1:1.1:1.0:0.9:1.3. **Pronotum** subtrapezoidal, finely punctured, anterior margin nearly straight, rounded at sides, narrower than base, basal margin widely bisinuate, disc moderately convex at apical 2/3, weakly depressed in middle of each half of basal 1/3 (Fig. 1a, Fig. 2b). Scutellum tongue-shaped, rounded apically and concave in middle. **Elytra** slightly wider than pronotum, about 3.1 times as long as wide, parallel-sided from base to apical 1/4, then gradually narrowed apically. First elytral band short, transverse, oval or rectangle-shaped with rounded edges, in middle of basal 1/7 of each elytron; second band at outer half at basal 1/4 of elytron, transverse, sometimes nearly caret-shaped or slightly oblique backwards; third band in middle of elytron, transverse, nearly reaching suture, thickening quickly and rounded at inner end; fourth band at apical 1/4 of elytron, oblique at inner 2/3 or transverse. Though length and shape of four elytral bands variable among individuals, i.e. length of second band, width of third band and curveness of fourth band, these four bands are well separated, none of them connected to any other band (Fig. 1a, c and d). Pale pubescence at apical 1/7 of elytra, not dense as elytral bands, not reaching fourth elytral band. Ventral side sparsely and finely punctured. Median portion of prosternum short. Median portion of apical mesosternum with sparse granules and a median ridge (Fig. 1b, i), pointed apically. Mesosternal process long and acute, but not dividing mesocoxae completely. Posterior half of metasternum with a groove, apical of median metasternum projected and pointed on both sides of the groove (Fig. 1b, ii). Abdomen sparsely and finely punctured, 5^th^ sternite slightly concave in middle at apex.

**Figure 3.**
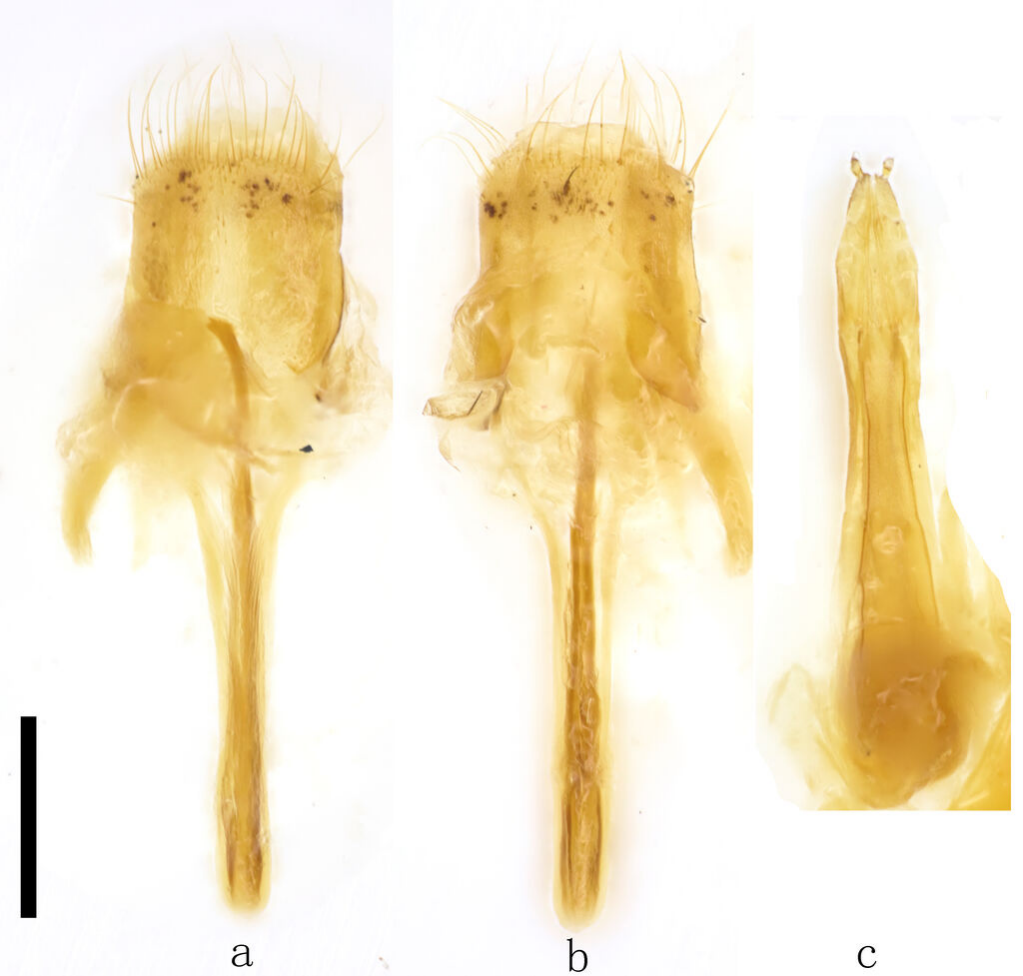
Female genitalia of *Stenoxylitaquadrifasciata* sp. nov. a, b. Abdominal segment VIII and spiculum ventrale; c. ovipositor (a, c. ventral view; b. dorsal view; scale bar, 0.5 mm).

Female genitalia. Sternite VIII and tergite VIII longer than wide, with sparse long setae mixed with several short setae apically (Fig. 3a, b), apex of sternite VIII broadly rounded, slightly concave in middle (Fig. 3a), apex of tergite VIII broadly rounded (Fig. 3b). Ovipositor slightly constricted in middle, apical half spear-shaped, basal half gradually widening toward base, width at base wider than maximum width of apical half (Fig. 3c).

Male unknown. Based on characters of its congeners and other melandryids, males may present only small morphological differences from females, except the dilated basal three tarsomeres of front tarsi.

#### Diagnosis

*Stenoxylitaquadrifasciata* sp. nov. differs from its congeners, *Stenoxylitatrialbofasciata* (Hayashi & Katö, 1956) and *Stenoxylitasasajii* Toyoshima, 2001, in terms of the number and pattern of elytral bands. As indicated by their specific names, the new species exhibits four bands on each elytron, whereas *S.trialbofasciata* only has three bands on each elytron. Basal half of pronotum and scutellum of *S.trialbofasciata* are dark red, whereas pronotum and scutellum are black in *Stenoxylitaquadrifasciata* sp. nov. In *S.sasajii*, the first elytral band is arcuate, with the sutural end reaching the scutellum, whereas in *Stenoxylitaquadrifasciata* sp. nov., it is short and transverse, located away from the scutellum. Additionally, the second band in *S.sasajii* bends obliquely backwards and reaches the third band near the inner end of the third band, while, in *S.quadrifasciata* sp. nov., the second band is short and transverse at the outer half of elytron, not extending backwards nor reaching the third band in any of availabe nine specimens (Fig. 1a, c and d).

#### Etymology

The specific name of this species refers to the four pale bands on each elytron.

## Supplementary Material

XML Treatment for Stenoxylita quadrifasciata
